# The Role of Natural Killer Group 2, Member D in Chronic Inflammation and Autoimmunity

**DOI:** 10.3389/fimmu.2018.01219

**Published:** 2018-05-30

**Authors:** Marina Babic, Chiara Romagnani

**Affiliations:** ^1^Innate Immunity, German Rheumatism Research Center (DRFZ), Leibniz Association, Berlin, Germany; ^2^Medical Department I, Charité – Universitätsmedizin Berlin, Berlin, Germany

**Keywords:** autoimmunity, natural killer group 2, member D, natural killer group 2, member D ligand, intestinal inflammation, rheumatoid arthritis, multiple sclerosis, type 1 diabetes

## Abstract

Current medicine and medical science puts great effort into elucidating the basis of chronicity and finding appropriate treatments for inflammatory diseases; however, the mechanisms driving aberrant immune responses are mostly unknown and deserve further study. Of particular interest is the identification of checkpoints that regulate the function and differentiation of pro-inflammatory cells during pathogenesis, along with means of their modulation for therapeutic purposes. Natural killer group 2, member D (NKG2D) is a potent activator of the immune system, known as a sensor for “induced-self” ligands, i.e., cellular danger signals that, in the context of chronic inflammation and autoimmunity, can be presented by cells being exposed to an inflammatory cytokine milieu, endoplasmic reticulum stress, or cell death. Engagement by such ligands can be translated by NKG2D into activation or co-stimulation of NK cells and different subsets of T cells, respectively, thus contributing to the regulation of the inflammatory response. In this review, we discuss the current knowledge on the contribution of the NKG2D–NKG2DL signaling axis during intestinal inflammation, type 1 diabetes, multiple sclerosis, and rheumatoid arthritis, where the role of NKG2D has been associated either by aberrant expression of the receptor and its ligands and/or by functional data in corresponding mouse models.

## Introduction

Natural killer group 2, member D (NKG2D; encoded by the gene *Klrk1*) is a molecular sensor of stressed cells and a potent activator of the immune system, and is largely expressed by NK cells as well as CD8^+^ and γδ^+^ T cells (1). It binds to a variety of well-defined danger molecules, such as retinoic acid inducible early (RAE) 1α–ε, H60a–c and murine UL-16-binding protein-like transcript (MULT)-1 in mice, or the MHC class-I-related chain (MIC)A/B and the UL-16 binding proteins (ULBP)1–6 in humans (2). The transcripts of NKG2D ligands are found in many tissues under healthy conditions but the cell surface expression of the corresponding proteins is kept under control by post-transcriptional regulation (3). Cellular stress, notably DNA damage, toll-like receptor signaling, and specific cytokine exposure can induce NKG2D ligand surface expression, as shown on tumor or virally infected cells (2). Ligand recognition by NKG2D can be integrated into a DNAX-activating protein (DAP)12- or DAP10-dependent signal, as in activated mouse NK cells, or into a DAP10-PI3K-mediated killing signal in human NK cells as well as a co-stimulation signal in human and mouse CD8^+^ T cells (4–6). Based on this, NKG2D plays an important role in immune surveillance by mediating direct recognition and clearance of infected and transformed cells expressing cognate ligands (1, 7, 8). However, upregulation of NKG2D ligands has also been reported in tissue samples from patients with chronic inflammatory and autoimmune disorders as well as in *in vivo* experimental models thereof, and NKG2D^+^ cells have been implicated in their pathogenesis. Interestingly, in addition to NK, CD8^+^ T, and γδ^+^ T cells, increased frequency of rarely occurring NKG2D^+^CD4^+^ T cells has been observed in many different human pathologies (9–12) and in inflammatory models in mice (13, 14).

Here, we discuss the role of NKG2D-expressing cells as well as of NKG2D ligands during selected chronic inflammatory diseases, where the aberrant expression of the receptor and its ligands or data in corresponding mouse models implicate the role of NKG2D in the development of the respective disease.

## Celiac and Inflammatory Bowel Diseases (IBDs)

Celiac disease (CeD) is a malabsorption syndrome that is elicited by gluten intolerance in individuals with genetic susceptibility. The pathology is manifested in massive cell death in the epithelial compartment which is infiltrated by autoreactive cytotoxic lymphocytes (CTL). The disruption in the homeostasis of intraepithelial lymphocytes (IEL) can additionally lead to the development of lymphoid malignancies, often associated with the refractory celiac sprue (RCS) (15), caused either by the clonal expansion of T cells or lately characterized sCD3^−^iCD3^+^ innate IEL (16). Patients with active disease are characterized by high levels of IL-15 expressed by intestinal enterocytes and lamina propria (LP) mononuclear cells, which correlates with the degree of mucosal damage (17).

IL-15 seems to mediate priming of CD8^+^ T cells and turns them into potent cytolytic cells (18), which kill epithelial cells based on the recognition of stress signals (19). It became evident that IL-15 contributes to the cytotoxic potential of CTL by increasing the expression of NKG2D and its adaptor DAP10 in CD8^+^ T cells, and indeed, patients with active CeD display a 4- to 20-fold higher expression of NKG2D on intraepithelial CTL, when compared with healthy individuals (18). Importantly, the expression of NKG2D ligands, namely, MICA, is upregulated in intestinal epithelial cells (IEC) as well as in mononuclear cells of patients. NKG2D blocking inhibited lysis of MIC^+^ or ULBP^+^ IEC lines (or of MICA-transfected tumor cell lines) by IL-15-primed human effector CTL, suggesting that upregulation of NKG2D on CTL converts them into potent killers (18). Conversely, the role of NKG2D expression by other T cell subsets or by innate lymphoid cells (ILC) during CeD has not been investigated so far.

The crucial role of gluten in the pathogenesis of CeD is also partially linked to the regulation of the NKG2D pathway. Interestingly, upon transition of patients to a gluten-free-diet (GFD), the levels of both NKG2D and MICA dropped, reaching levels observed in healthy controls, but remained high in GFD-resistant RCS patients. Moreover, culturing intestinal samples with gliadin, resulted in the upregulation of MICA exclusively in patients on a GFD, but not in healthy controls. Upon testing of different gliadin-derived peptides, non-immunodominant p31–49 and immunodominant p57–89, it became clear that only p31 induced MICA expression (20). p31–49 induced IL-15 expression by LP dendritic cells and macrophages of CeD patients (21), and the p31-mediated upregulation of MICA could be blocked by α-IL-15 neutralizing antibodies (20), suggesting that this might be a mechanism of how the sensing of gluten and IL-15 is integrated to result in the expression of NKG2D and MICA and contribute to CeD pathogenesis. Despite these interesting observations, data reporting on NKG2D or MIC polymorphisms in association with susceptibility to CeD are either absent or conflicting (20, 22, 23) and the demonstration of a functional role of NKG2D in an *in vivo* CeD experimental model is still missing to date.

Inflammatory bowel disease represents a group of intestinal disorders that cause prolonged inflammation of the digestive tract and is prominently represented by Crohn’s disease (CD) and ulcerative colitis (UC). IBD is characterized by dysregulated gut microbiota, and an aberrant immune response, typically dominated by Th1 and Th17 cells during CD and Th2 cells during UC (24, 25). Association of the NKG2D–NKG2DL axis in the pathogenesis of IBD was implicated after the discovery of significantly upregulated MICA expression in IEC from CD and to a lesser extent from UC patients when compared with IEC from area-matched healthy controls (9). However, characterization of CD4^+^ T cells from the peripheral blood (PB) and LP of CD and UC patients or control individuals revealed an increase in the frequency of NKG2D^+^ cells exclusively in CD patients. These cells displayed a Th1-like phenotype, reflected in perforin expression, secretion of IFN-γ upon stimulation and cytotoxicity toward MICA-expressing targets (9). NKG2D^+^CD4^+^ T cells from LP of CD patients expressed IL-15Rα and IL-15 provision increased NKG2D and DAP10 expression in CD4^+^NKG2D^+^ clones, similarly as described for CD8^+^ T cells in CeD. Whether IL-15 is the main and only factor driving NKG2D expression on CD4^+^ T cells remains to be determined. Following the discovery of Th17 cells and their role in mediation of intestinal inflammation, further profiling of NKG2D^+^CD4^+^ T cells from CD patients showed enrichment of IL-17-producing cells among NKG2D^+^CD4^+^ T cells when compared to the NKG2D^−^ compartment. Co-stimulation of CD4^+^ T cells *via* NKG2D resulted in expression of IL-17, IFN-γ, and TNF. IL-17A/IFN-γ and IL-17A/IL-22 co-producers were specifically contained within NKG2D^+^CD4^+^ T cells (26). T-cell receptor repertoire analysis showed that most CD4^+^ T cell oligoclonal expansions found in PB and small intestine LP of CD patients are contained within the NKG2D^+^ subset (27). Interestingly, the expansions found in LP and PB were different, suggesting that the ones in the LP are a consequence of a local expansion. Two separate studies demonstrated accumulation of NKG2D^+^CD4^+^ T cells in colon LP in a CD4^+^ T cell transfer-induced colitis model and disease amelioration after treatment with blocking α-NKG2D antibodies (13, 28).

Using a model of dextran sulfate sodium (DSS)-induced colitis, Qian and colleagues reported perturbation of numbers and frequency of NKG2D^+^CD4^+^ and NKG2D^+^CD8^+^ T cells in colon and spleen (29). Further dissection of splenic NKG2D^+^CD4^+^ T cells according to NK1.1 expression revealed two major subsets, namely, TGF-β^+^FasL^+^T-bet^+^NK1.1^−^ cells and IFN-γ^+^IL-17^+^IL-21^+^granzymeB^+^perforin^+^T-bet^−^RORγt^+^NK1.1^+^ cells. Transfer of NK1.1^−^NKG2D^+^CD4^+^ T cells delayed the onset of DSS-induced colitis and the protective effect was dependent on TGF-β. Conversely, the transfer of NK1.1^+^NKG2D^+^CD4^+^ T cells exacerbated the colitis outcome.

An interesting study by Hosomi and colleagues demonstrated an important role of the NKG2D–NKG2DL interaction in recognition of endoplasmic reticulum stress by immune cells and how this is converted into intestinal inflammation (30). Mice with specific deletion of *Xbp1*, a crucial gene involved in unfolded protein response in IEC (*Xbp1*^ΔIEC^) showed increased expression of *Ulbp1* (gene encoding MULT-1) in IEC, as well as spontaneous development of intestinal inflammation. This process seems to be mediated by direct binding of the transcription factor, named CCAAT-enhancer-binding protein homologous protein (CHOP), to the promotor region of *Ulbp1* in IEC. Spontaneous enteritis was ameliorated by treatment with an α-NKG2D-blocking antibody as well as by depletion of NK1.1^+^ cells that include cytolytic NKG2D-expressing group 1 ILC. Despite increased frequency of intraepithelial NKG2D^+^γδ^+^ T cells observed in *Xbp1*^ΔIEC^ mice, γδ^+^ or αβ^+^ T cells play a redundant role in causing spontaneous intestinal inflammation in this model (30). Altogether, these reports show that upregulation of NKG2D ligands might represent a common response to intestinal epithelium stress, rendering IEC susceptible to NKG2D-mediated immune surveillance and regulation.

## Type 1 Diabetes (T1D)

During T1D, the body’s own immune system attacks the β-cells in the pancreatic islets resulting in damage, reduced and subsequently abrogated insulin production (31). Genetically susceptible individuals carry the high-risk HLA DR4-DQ8 and DR3-DQ2 haplotype in more than 90% of cases. Although it is generally believed that the disease is mediated by self-reactive CD8^+^ and CD4^+^ T cells and macrophages, a role for regulatory T cells in the regulation of diabetogenic IFN-γ-producing NK cells in the pancreatic islets has been described (32, 33). In humans, genetic linkage studies showed positive association of the MICA allele 5 with T1D (34). The role of NKG2D in T1D pathogenesis has been assessed mainly using the non-obese diabetic (NOD) mice, with conflicting conclusions (35–37). An initial study reported *Raet1* transcripts (encoding for RAE1) in the β-cells of the pancreas of 4- to 6-week-old NOD mice and linked an increase in *Raet1* expression with age (36). Following studies could not confirm the expression of *Raet1* in pancreatic β-cells of NOD mice (37–39), while Trembath et al. rather observed expression of *H60a* in pancreas-infiltrating T cells (37). A pathogenic role for NKG2D was reported by Ogasawara et al. (36), who demonstrated that antibody-mediated blocking of NKG2D signaling led to reduced infiltration of autoreactive CD8^+^ T cells into the pancreas of 16-week-old NOD mice and decreased diabetes incidence. Along this line, by using the C57BL/6J mice with transgenic expression of RAE1ε in islet β-cells of the pancreas (*Rae1*-Tg mice), it was shown that CTL were recruited to the pancreas of *Rae1*-Tg mice in an antigen-independent but NKG2D-dependent manner (40). Although transgenic expression of RAE1 led to spontaneous insulitis in old *Rae1*-Tg mice, no diabetes development could be observed in this model. Disease amelioration by antibody-mediated blocking of NKG2D signaling in NOD mice, as initially reported by the Lanier group (36), could not be reproduced in the report by Guerra et al. (35). In addition, crossing of NKG2D-deficient mice to the NOD background (NOD × *Klrk1*^−/−^) did not result in any disease amelioration (35), questioning the role of NKG2D in this disease model.

One potential explanation of such discrepancies can be related to microflora differences, which appear to impact T1D incidence in NOD mice (41, 42). Interestingly, Trembath and colleagues reported that *Klrk1*^−/−^ NOD mice have lower diabetes incidence when compared with littermate NOD mice housed in specific pathogen-free conditions; however, this effect was lost and even reversed upon treatment of NOD and *Klrk1*^−/−^ NOD littermate mice with broad-spectrum antibiotics (37). Supporting this hypothesis, it was shown that treatment of C57BL/6N mice with vancomycin can reduce the expression of *Raet1* in small intestine epithelium, and this effect was correlated with the presence of *A. muciniphila* in the vancomycin-treated mice (43).

In light of these data, the role of NKG2D in T1D autoimmunity should be reconsidered by taking into account the differences in colonizing microbiota. In addition, the contribution of other NKG2D-expressing cell types to the regulation of T1D needs further evaluation.

## Multiple Sclerosis (MS) and Experimental Autoimmune Encephalomyelitis (EAE)

Multiple sclerosis, an inflammatory disease of the central nervous system (CNS), is characterized by the loss of oligodendrocytes, followed by a reduction in myelin production. While CD4^+^ T cells predominate in acute lesions of MS patients and are better studied in the EAE, a mouse model of MS (44), CD8^+^ T cells seem to play an important role in tissue damage and are observed more frequently in the chronic lesions of MS patients (45, 46). Patients display elevated levels of soluble MICB but not MICA in the sera (47) and the MICB*004 allele was associated with higher MS susceptibility (48), suggesting a role for NKG2D signaling in the development of MS. Similar to other inflammatory diseases, IL-15 was elevated in the serum of MS patients and could be expressed *ex vivo* in astrocytes and infiltrating macrophages from MS lesions (49). Astrocyte-derived IL-15 reinforced the cytotoxic program in CD4^+^ and CD8^+^ T cells, with increased expression of NKG2D, perforin, and granzyme (49, 50). MICA/B ligand engagement on human oligodendrocytes could induce their killing *in vitro* by IL-2 activated NK cells, γδ^+^ T cells, or polyclonal CD8^+^ T cells in an NKG2D-dependent fashion (50).

Multiple sclerosis patients are characterized by an enrichment in NKG2D^+^CD4^+^ but not CD8^+^ T cells in cerebrospinal fluid and in inflammatory lesions (51), as compared to PB and to healthy donors, which might be a result of a local expansion mediated by an inflamed milieu or an increased migratory capacity (49). In the same study, by using the EAE model, the authors could show that treatment of mice with a blocking α-NKG2D antibody after immunization, but before disease onset, resulted in a reduced disease score (51), which could partially be reproduced by using *Klrk1*^−/−^ mice (35). The treatment did not seem to affect peripheral cell activation, but rather the infiltration of NKG2D^+^CD4^+^ and NKG2D^+^CD8^+^ T cells into CNS at the peak of the disease. Moreover, *Raet1* transcripts were detected in myeloid cells in the spinal cord of the EAE mice, with expression levels correlating with the disease score (52). It was suggested that NKG2D^+^CD4^+^ T cells might contribute to killing of oligodendrocytes. Similar to human, *in vitro* cultured and cytokine activated mouse oligodendrocytes expressing MULT-1 and RAE1 were susceptible to killing by α-CD3/α-CD28 activated CD4^+^ T cells enriched for the expression of NKG2D to 8–12% (51). Although the killing mechanism remains unclear, it seems to be partially reduced by blocking of NKG2D, and independent of MHC class I or II peptide presentation.

While accumulation of NKG2D^+^CD4^+^ T cells into CNS of EAE and MS remains an interesting finding, the contribution of NKG2D on various cell types in mediating cell priming in the periphery, migration into the tissue and reactivation *in situ* needs to be further explored.

## Rheumatoid Arthritis (RA) and Models of Joint Inflammation

Rheumatoid arthritis is a chronic inflammatory disease that causes inflammation and destruction of the joints. The disease is characterized by high levels of TNF and IL-15 which are found in patient sera (10) and prominently mediated by joint-infiltrating autoreactive CD4^+^ T cells that promote autoantibody production by plasma cells, along with macrophage and endothelial activation (53). It was shown that TNF and IL-15 could upregulate NKG2D *in vitro* on PB CD4^+^ T cells or on NKG2D^−^CD4^+^ T cells from the inflamed synovia of RA patients. Indeed, around 18% of potentially autoreactive CD28^−^CD4^+^ T cells from the PB and synovial tissue of RA patients expressed NKG2D. Membrane bound MICA and MICB were abundantly expressed in the synoviocytes of RA patients and could trigger autologous autoreactive T cells in an NKG2D-dependent manner (54). Crosslinking of NKG2D on CD4^+^CD28^−^ T cells seemed to co-stimulate TCR-mediated secretion of IFN-γ and TNF as well as proliferation (10).

Single-nucleotide polymorphisms in both MICA and NKG2D have been associated with RA, suggesting MICA and NKG2D as RA susceptibility genes (55, 56). Moreover, a recent study evaluated the role of polymorphisms in *KLRK1* gene with efficacy of α-TNF therapy in RA patients (57), identifying two polymorphisms associated with better response and two polymorphisms associated with inefficient response to therapy. More functional studies that would dissect the direct effect of the polymorphisms to the TNF production and resistance to α-TNF therapy would be beneficial to understand the impact of this association.

Using a collagen-induced arthritis (CIA) model, Andersson and colleagues could demonstrate that the treatment of mice with a blocking α-NKG2D antibody reduced the clinical score, even when applied after the disease onset (14). Along this line, NKG2D-blocking preserved joint architecture and reduced infiltration of γδ^+^ and CD4^+^ T cells in mouse paws, while it did not affect CD8^+^ T and NK cells. This study also reported that treatment with an α-NKG2D-blocking antibody reduced infiltration of IL-17^+^CD4^+^ but not of IL-17^+^γδ^+^ T cells in the paws. Interestingly, the per-cell expression of NKG2D on NK cells was slightly reduced during late CIA, possibly related to chronic exposure of these cells to the ligands. Similar to what was observed in human RA samples, NKG2D ligand expression did not affect the expression of NKG2D on γδ^+^ and CD4^+^ T cells, suggesting different regulation of NKG2D expression on innate and adaptive cells.

## Concluding Remarks

Current data support the role of inflammatory cytokine- and endoplasmic reticulum stress-induced expression of NKG2D ligands in several immune-mediated diseases, suggesting that the NKG2D–NKG2DL axis can represent an interesting target for the modulation of selective inflammatory disorders. Recently, the efficacy of a blocking α-NKG2D antibody was tested within phase I/II clinical trial that included 78 patients diagnosed with CD. No difference between the single dose treated and placebo receiving cohort was observed at week 4, but effects became visible at week 12 as manifested in a significant clinical response in patients responding to biological therapy or those with untreated disease (58). A phase I/II study with a blocking α-NKG2D antibody was also performed in a cohort of patients diagnosed with RA, however, with no published results to date. The field would benefit from functional studies performed *in vivo*, which would allow comparisons between complete NKG2D-deficient mice to mice with cell-specific NKG2D-deficiency (59), as well as from studies in mice with conditional deficiency of NKG2D ligands (60). Given the complexity of the regulation of NKG2D ligand expression and the possibly multifaceted role of microbiota, understanding the specific role of each NKG2D^+^ population in the pathogenesis of the particular disease would enable better design of future therapeutic approaches.

## Author Contributions

MB searched the literature and wrote the majority of the manuscript. CR contributed to writing and editing of the manuscript.

## Conflict of Interest Statement

The authors declare that the research was conducted in the absence of any commercial or financial relationships that could be construed as a potential conflict of interest. The reviewer DA and handling Editor declared their shared affiliation.
